# Health systems perspectives – infectious diseases of poverty

**DOI:** 10.1186/2049-9957-1-12

**Published:** 2012-11-01

**Authors:** Dale Huntington

**Affiliations:** 1 Asia Pacific Observatory on Health Systems and Policies, WHO/WPRO, Manila, Philippines

## Abstract

The right to health as a fundamental human right is enshrined in the World Health Organization’s charter and has been reaffirmed in international agreements spanning decades. This new journal reminds us of the essential characteristic of poverty as a violent abuse of human rights. The context of poverty – its social, political and economic dimensions – remain in the reader’s mind as evidence is provided on technical solutions to managing the infectious diseases that afflict poor populations world-wide. Applying a health systems framework to a discussion on infectious diseases of poverty emerges from the papers in this journal’s first edition. Many of the articles discuss treatments, indicating the importance of pharmaceuticals for neglected diseases. Delivery strategies to reach impoverished populations also figure within this first round of papers. Innovative programs that provide diagnostics and treatment for infectious diseases to hard-to-reach rural and urban communities are needed clearly needed, and some good examples are discussed here. Future editions will explore other health system components, broadening the evidence base to increase understanding of effective and sustainable interventions to reduce the burden of infectious disease among the poor. The editors are to be congratulated on the release of this inaugural issue of the journal Infectious Diseases of Poverty. We look forward to reading subsequent editions.

## Multilingual abstracts

Please see Additional file 1 for translations of the abstract into the six official working languages of the United Nations.

## Commentary

“When we assess inequalities…in being able to avoid preventable morbidity, or escapable hunger, or premature mortality, we are not merely examining differences in well-being…(T)he available data regarding the realization of disease, hunger and early mortality tell us a great deal about the presence or absence of certain central basic freedoms [1].”

The right to health as a fundamental human right is enshrined in the World Health Organization’s charter and has been reaffirmed in international agreements spanning decades. This new journal reminds us of the essential characteristic of poverty as a violent abuse of human rights. The context of poverty – its social, political and economic dimensions – remain in the reader’s mind as evidence is provided on technical solutions to managing the infectious diseases that afflict poor populations world-wide [2]. Explorations into these political economic factors that increase the risk of the poor for certain diseases are beyond the scope of this journal, as its focus remains more heavily centered on public health aspects of managing infectious diseases. Yet its title and intended scope are reminders of the extreme inequalities that exist between and within countries worldwide, with devastating consequences for human and economic development.

The past decade’s Commission on the Social Determinates of Health provided a pathway across the social science and public health disciplines, and remains a useful source of good thinking on a set of complex issues [3]. Today health policy is currently being framed in terms of achieving universal health coverage [4]. Although financing is a major element of universal health coverage (through reducing financial barriers to needed health care and ensuring an adequately funded public health system), other health system elements are as important: human resources, service delivery sites, medical supplies and equipment.

A useful beginning to applying this health systems approach to a discussion on infectious diseases of poverty is to examine key aspects of each system component [5]. Many of the articles in this journal’s first volume discuss treatments, indicating the importance of pharmaceuticals for neglected diseases [6-8]. There are a number of issues with the development, pricing and manufacturing of pharmaceuticals related to infectious diseases of poverty that we look forward to reading in future editions.

Delivery strategies to reach impoverished populations also figure within this first round of papers [9]. Innovative programs that provide diagnostics and treatment for infectious diseases to hard-to-reach rural areas are needed [10]. Problems with access are not just geographic, but can also be caused by social barriers, particularly in urban areas [11-13]. The scope of work that is to be discussed in the journal should include both public and private sector delivery systems. For example, social franchising networks are growing worldwide with the aim to serve the poor, and commonly include treatments for infectious diseases.

Human resources are closely related to service delivery strategies: The deployment of skilled health workers in areas of endemic poverty is a ubiquitous problem that health systems in every country world-wide (no matter what level of economic development) are struggling with. Health workers are needed for preventive care services in addition to treatment [9]. Health education, behavioral change and communication, and community-based development programs as they relate to infectious diseases of poverty all fall within the scope of this new journal. We look forward to reading of new applications of tried and true programs, as well as innovative use of social media, mobile phone and other new electronic platforms for improving health of poor populations.

Topics of health financing systems, including the growth of social health insurance schemes are directly relevant to a journal on infectious diseases of poverty [14]. Financing topics should include discussions of resource flows into national health budgets for infectious diseases of poverty. Papers that have a health economics focus will be good reading from this journal, including perspectives on operations of global funding platforms, e.g., forthcoming changes in the Global Fund against TB, Malaria and HIV/AIDS [12].

**Figure 1:**
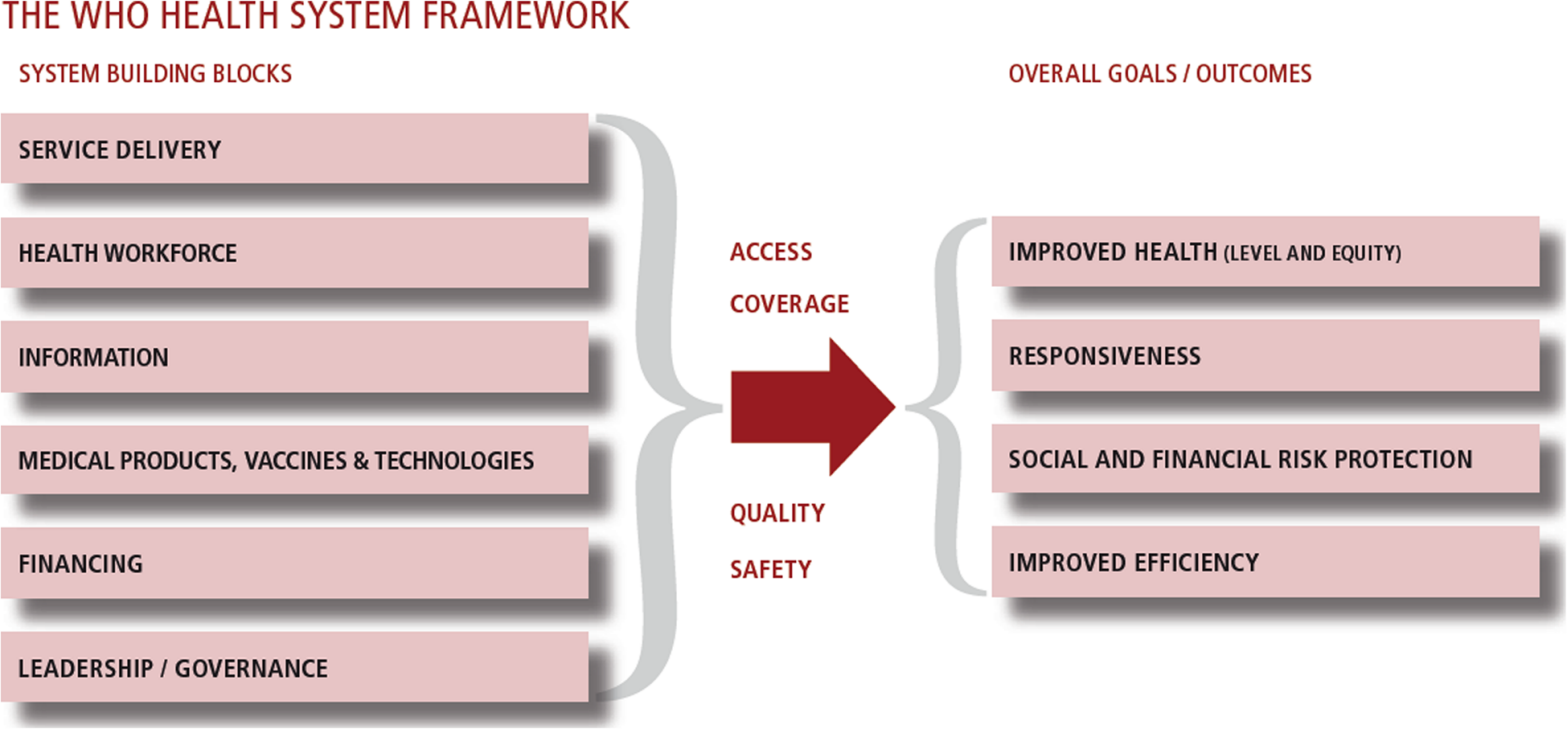
**Health Systems Building Blocks.** (Source: WHO Health Systems Strategy, http://www.who.int/healthsystems/strategy/en/).

There are many other health system component topics that could be listed, as suggested in the figure below, but for reasons of space I’ll stop with these few illustrative examples. Indeed, it will be challenging to establish boundaries to some of these themes – just as the intention to expand the publication beyond efficacy of treatment. Infectious diseases of poverty are not limited to the three diseases targeted by the Global Fund against TB, Malaria and HIV/AIDS, as suggested by some of the articles in this issue [2,6]. The editors are to be congratulated on the release of this inaugural issue of the journal Infectious Diseases of Poverty. We look forward to reading subsequent editions (Figure 1). 

## Competing interest

I declare that I have no competing interests.

## Supplementary Material

Additional file 1 Multilingual abstracts in the six official working languages of the United Nations. Click here for file
