# Supplementary material for: Health systems perspectives – infectious diseases of poverty
Source: Infect Dis Poverty. 2012 Nov 1;1:12. doi: 10.1186/2049-9957-1-12 (PMC3710094; doi:10.1186/2049-9957-1-12)
Supplement: Additional file 1 — Multilingual abstracts in the six official working languages of the United Nations. [file 2049-9957-1-12-S1.pdf]

Translation of the abstract into the six official working languages of the United Nations

ديل إي هنتنغتون

الملخص

الحق في الصحة أمر مكرس كحق أساسي من حقوق الإنسان في ميثاق منظمة الصحة العالمية، وقد تم التأكيد عليه في الاتفاقات الدولية الممتدة منذ عقود. هذه المجلة الجديدة تذكرنا بالسمات الأساسية للفقر باعتباره انتهاك عنيف لحقوق الإنسان. مشكلة الفقر - بأبعادها الاجتماعية والسياسية والاقتصادية - تبقى في ذهن القارئ كدليل مقدم للحلول التقنية للتعامل مع الأمراض المعدية التي تصيب السكان الفقراء في جميع أنحاء العالم. تطبيق إطار النظم الصحية لمناقشة الأمراض المعدية التي يسببها الفقر يمكن استشفافها من مقالات أول أعداد هذه المجلة. تناقش العديد من هذه المقالات العلاجات، مشيرة إلى أهمية الأدوية للأمراض المهملة. استراتيجيات التوصيل إلى السكان الفقراء تتمثل أيضا في هذه المجموعة الأولى من المقالات. من الواضح أن هناك حاجة لبرامج مبتكرة تتمكن من توفير وسائل التشخيص والعلاج للأمراض المعدية للمجتمعات الريفية والحضرية التي يصعب الوصول إليها، وهناك مناقشة لبعض الأمثلة الجيدة هنا. ستحاول الأعداد المقبلة استكشاف المكونات الأخرى للنظام الصحي، وتوسيع قاعدة الأدلة لزيادة فهم التدخلات الفعالة والمستدامة للحد من عبء الأمراض المعدية بين الفقراء. يستحق المحررون التهنئة لإصدارهم هذا العدد الافتتاحي من مجلة الأمراض الفقر المعدية. ونحن نتطلع قدما إلى قراءة الأعداد اللاحقة.

Translated from English version into Arabic by Lina SM, through

TRANSLATORS
WITHOUT BORDERS

卫生体制展望——贫困所致传染病

Dale E Huntington

摘要

健康权作为一项基本人权已载入世界卫生组织章程，几十年来经国际协议再次确认。这本新杂志让我们想起贫困的本质特征如同暴力侵犯人权。在贫困的背景下，即其社会、政治和经济方面在读者脑海中的印象，是为危害世界各地贫困人口传染病的管理提供技术解决方案。本刊第一期的文章对适用于贫困所致传染病的卫生体制的框架展开了讨论。许多文章探讨了治疗方法，说明了被忽视疾病药物应用的重要性。第一期还包括将服务递传策略用于贫困人口的论文。明确需要为难以抵达的农村和城市社区提供诊断和治疗传染病的创新项目，在本刊对一些很好的案例进行了讨论。未来几期杂志将探索其他卫生体制组成部分，进一步扩大以减少穷人传染病负担的有效和可持续措施的证据。我们将庆祝本刊的首刊发布，并期待阅读后续期刊。

Translated from English version into Chinese by Yang Pin, through

CDC CHINESE CENTER FOR DISEASE CONTROL AND PREVENTION
NATIONAL INSTITUTE FOR PARASITIC DISEASES

Perspectives sur les systèmes de santé – maladies infectieuses dues à la pauvreté

Dale E Huntington

Résumé

En tant que droit fondamental de l'homme, le droit à la santé est inscrit dans la Charte de l'Organisation mondiale de la Santé et a été réaffirmé dans les accords internationaux depuis des décennies. Cette nouvelle revue nous rappelle la caractéristique essentielle de la pauvreté en tant que profonde violation des droits de l'homme. En effet, le contexte de la pauvreté (sa dimension sociale, politique et économique) reste présent dans l'esprit du lecteur lorsqu'on propose des solutions techniques pour gérer les maladies infectieuses dont souffrent les personnes pauvres dans le monde. L'application d'un cadre pour les systèmes de santé à un débat sur les maladies infectieuses dues à la pauvreté ressort des articles de cette première édition. La majorité d'entre eux portent sur le traitement, ce qui indique l'importance des produits pharmaceutiques pour les maladies négligées. Les stratégies de distribution pour atteindre les populations pauvres figurent également dans ces articles. D'autre part, les programmes novateurs qui fournissent des diagnostics et traitements aux communautés rurales et urbaines difficiles d'accès sont une nécessité absolue, et quelques exemples représentatifs sont présentés ici. Les prochaines éditions exploreront les autres composantes du système de santé, permettant ainsi d'élargir la base de connaissances pour une meilleure compréhension des interventions durables et efficaces afin de réduire le fardeau que représentent les maladies infectieuses auprès des personnes pauvres. Les éditeurs sont à féliciter pour cette première parution de la Revue sur les maladies infectieuses dues à la pauvreté. Nous attendons avec impatience de lire les prochains numéros.

Translated from English version into French by Essaka_j, through

TRANSLATORS WITHOUT BORDERS

Перспективы развития систем здоровья - инфекционные заболевания бедности

Дейл Э. Хантингтон

Тезисы

Право на здоровье является фундаментальным правом человека, зафиксированным в своде Мировой Организации Здоровья и подтверждаемым международными соглашениями в течение многих десятилетий. Этот журнал напоминает о том, что бедность - это жестокое нарушение прав человека. Контекст бедности - ее социальные, политические и экономические подоплеки, - остаются неоспоримыми по мере того, как появлются новые технологические решения по контролю инфекционных заболеваний, угрожающих бедному населению мира. Применение рамок систем здоровья к обсуждению инфекционных заболеваний бедности было впервые предпринято в этом первом издании журнала. Многие статьи фокусируются на лечении, указывая на важность использования фармацевтических продуктов для борьбы с запущенными болезнями. Стратегии доставки этих самых фармацевтических продуктов для бедных слоев населения также фигурируют среди первых статей в журнале. Инновативные программы, рассматривающие способы диагностирования и лечения инфекционных заболеваний в труднодоступных деревенских и городских районах, крайне необходимы, и здесь обсуждаются некоторые тому примеры. Будущие издания сфокусируются на других проблемах систем здоровья, выводя на передний план больше показательных примеров, чтобы показать способы эффективного и устойчивого воздействия для сокращения ущерба от инфекционных заболеваний среди бедного населения. Мы должны выразить благодарность издателям за работу над этим первым изданием журнала “Инфекционные заболевания бедности” - и надеемся увидеть выход последующих выпусков.

Translated from English version into Russian by Elanorielle, through

TRANSLATORS
WITHOUT BORDERS

Perspectivas de los Sistemas Sanitarios- Enfermedades Infecciosas de Pobreza

Dale E Huntington

Resumen

El derecho a la salud como un derecho fundamental del ser humano está consagrado en los estatutos de la Organización Mundial de la Salud y se ha reafirmado en acuerdos internacionales a lo largo de varias décadas. Esta nueva revista nos recuerda que la pobreza esencialmente se caracteriza por constituir una violenta violación de los derechos humanos. El contexto de la pobreza (su alcance social, político y económico) permanece en la mente del lector en la medida en la que se aportan indicios sobre soluciones técnicas para controlar las enfermedades infecciosas que padece la población pobre de todo el mundo. En las disertaciones de la primera edición de esta revista surge la aplicación de una estructura de sistemas sanitarios al debate sobre enfermedades infecciosas en la pobreza. Muchos de los artículos versan sobre tratamientos, indicando la importancia de los fármacos en las enfermedades desatendidas. En la primera ronda de disertaciones también figuran estrategias de entrega para llegar hasta las poblaciones empobrecidas. Claramente, se necesitan programas innovadores que ofrezcan diagnóstico y tratamiento de enfermedades infecciosas para comunidades rurales y urbanas de difícil acceso y aquí se tratan algunos ejemplos. En próximas ediciones se explorarán otros componentes de sistemas sanitarios, ampliando la base evidente de aumentar la comprensión de intervenciones efectivas y sostenibles para reducir las enfermedades infecciosas entre los pobres. Los editores merecen ser felicitados por el lanzamiento de este número inaugural de la revista Infectious Diseases of Poverty. Esperamos poder leer próximas ediciones.

Translated from English version into Spanish by Adriana Blas, through

TRANSLATORS
WITHOUT BORDERS
